# Whole-Genome Analysis of Bordetella pertussis MT27 Isolates from School-Associated Outbreaks: Single-Nucleotide Polymorphism Diversity and Threshold of the Outbreak Strains

**DOI:** 10.1128/spectrum.04065-22

**Published:** 2023-05-16

**Authors:** Kazunari Kamachi, Kentaro Koide, Nao Otsuka, Masataka Goto, Tsuyoshi Kenri

**Affiliations:** a Department of Bacteriology II, National Institute of Infectious Diseases, Tokyo, Japan; Post Graduate Institute of Medical Education and Research

**Keywords:** *Bordetella pertussis*, outbreak isolate, single-nucleotide polymorphism, SNP diversity, SNP threshold, whole-genome sequencing

## Abstract

Bordetella pertussis, the causative agent of whooping cough, can cause pertussis outbreaks in humans, especially in school-aged children. Here, we performed whole-genome sequencing of 51 B. pertussis isolates (epidemic strain MT27) collected from patients infected during 6 school-associated outbreaks lasting less than 4 months. We compared their genetic diversity with that of 28 sporadic isolates (non-outbreak MT27 isolates) based on single-nucleotide polymorphisms (SNPs). Our temporal SNP diversity analysis revealed a mean SNP accumulation rate (time-weighted average) of 0.21 SNPs/genome/year during the outbreaks. The outbreak isolates showed a mean of 0.74 SNP differences (median, 0; range, 0 to 5) between 238 isolate pairs, whereas the sporadic isolates had a mean of 16.12 SNP differences (median, 17; range 0 to 36) between 378 isolate pairs. A low SNP diversity was observed in the outbreak isolates. Receiver operating characteristic analysis demonstrated that the optimal cutoff value to distinguish between the outbreak and sporadic isolates was 3 SNPs (Youden’s index of 0.90 with a true-positive rate of 0.97 and a false-positive rate of 0.07). Based on these results, we propose an epidemiological threshold of ≤3 SNPs per genome as a reliable marker of B. pertussis strain identity during pertussis outbreaks that span less than 4 months.

**IMPORTANCE** Bordetella pertussis is a highly infectious bacterium that easily causes pertussis outbreaks in humans, especially in school-aged children. In detection and investigation of outbreaks, excluding non-outbreak isolates is important for understanding the bacterial transmission routes. Currently, whole-genome sequencing is widely used for outbreak investigations, and the genetic relatedness of outbreak isolates is assessed based on differences in the number of single-nucleotide polymorphisms (SNPs) in the genomes of different isolates. The optimal SNP threshold defining strain identity has been proposed for many bacterial pathogens, but not for B. pertussis. In this study, we performed whole-genome sequencing of 51 B. pertussis outbreak isolates and identified a genetic threshold of ≤3 SNPs per genome as a marker defining the strain identity during pertussis outbreaks. This study provides a useful marker for identifying and analyzing pertussis outbreaks and can serve as a basis for future epidemiological studies on pertussis.

## INTRODUCTION

Bordetella pertussis causes pertussis, commonly referred to as whooping cough. Pertussis is a highly contagious respiratory disease that affects individuals of all age groups; however, its manifestation is particularly severe in infants. Although vaccination is the most effective method for preventing pertussis among infants, pertussis vaccines cannot provide long-term immunity (1–3). Therefore, pertussis outbreaks occur among individuals whose immunity has been compromised. Large outbreaks have occurred at the national and state levels over the last 2 decades (4–8), with small outbreaks occurring mainly in elementary and middle schools (9–11). In Australia, the United Kingdom, and the United States, large outbreaks were caused by several genetically distinct B. pertussis strains (12–14). In contrast, a single strain was usually detected in small outbreaks, such as school-associated outbreaks (15, 16).

Molecular strain typing of infectious agents is an important tool for epidemiological surveillance and outbreak investigations. Currently, whole-genome sequencing (WGS) is the most accurate method for genotyping; single-nucleotide polymorphism (SNP) typing (17, 18) and genome-wide multilocus sequence typing (core genome MLST and whole-genome MLST) are used for genotyping B. pertussis isolates (15, 19, 20). Whole-genome SNP analysis has the highest discriminatory power (21, 22) and is commonly used for detecting and investigating outbreaks. The genetic relatedness of isolates is assessed by the difference in the number of SNPs in the genomes of different isolates. For many bacterial pathogens, the optimal SNP threshold defining strain identity (i.e., defining isolates as the same outbreak strain) has been proposed: 2.5 SNPs for Acinetobacter baumannii (23), 5 SNPs for Escherichia coli O157:H7 and non-typhoidal Salmonella (24, 25), 5 or 12 SNPs, depending on epidemiological linkage, for Mycobacterium tuberculosis (26), 7 SNPs for vancomycin-resistant Enterococcus faecium (27), and 20 SNPs for Staphylococcus aureus (28). However, the SNP threshold for B. pertussis has not been reported yet.

In Japan, a nationwide pertussis outbreak occurred between 2008 and 2010, and small outbreaks occurred in rural areas in the 2010s (4, 11). Most small outbreaks occurred in elementary and middle schools, and multiple B. pertussis isolates were collected from patients during the school-associated outbreaks. The outbreak isolates were genotyped as the MT27 strain via classical multilocus variable-number tandem repeat analysis (MLVA); they were subtyped using simple SNP genotyping (16). B. pertussis MT27 is an epidemic strain recently reported worldwide (4, 12, 29, 30); thus, a sporadic MT27 strain not associated with an outbreak may also be isolated during outbreak investigations, especially in active microbiological surveillance (16). In outbreak investigations, excluding sporadic isolates is important for understanding bacterial transmission routes, and the SNP threshold defining strain identity plays a crucial role in genome-wide SNP analysis.

In the present study, we performed WGS of 51 B. pertussis MT27 isolates from 6 school-associated outbreaks and analyzed temporal SNP diversity in the isolates during the outbreaks. Further, we compared pairwise SNP differences among the outbreak isolates with those among 28 sporadic isolates. Moreover, we determined an optimal SNP threshold for distinguishing between outbreak and sporadic isolates during pertussis outbreak investigations.

## RESULTS

### Characteristics of the outbreak and sporadic isolates.

As shown in Table 1, the isolates were collected during six outbreaks that occurred during different periods. Fifteen isolates from outbreak 1 were collected within 110 days of the first sampling, and 10 isolates from outbreak 2 were collected within 30 days of the first sampling. The isolates from outbreaks 4, 5, 6, and 7 were collected within 89, 99, 36, and 13 days of sampling, respectively. The outbreak isolates of each outbreak revealed the same MLVA genotype, virulence-associated allelic genes, and 20-position SNP genotype. All outbreak isolates had the MLVA genotype MT27, which is common in the recent B. pertussis populations. The isolates from outbreaks 1, 2, 3, 4, and 5 carried the allelic profile *ptxP3*/*ptxA1*/*prn2*/*fim3A*, whereas the isolates from outbreak 6 harbored a distinct profile, *ptxP3*/*ptxA1*/*prn9*/*fim3A*. All isolates from outbreak 1 were classified as SG2 by simple SNP genotyping, and those from outbreak 5 were classified as SG5. The isolates from outbreaks 2 and 4 belonged to SG7, and those from outbreaks 3 and 6 belonged to SG10. Therefore, isolates corresponding to different outbreaks exhibited the same genotype and allelic profile.

**TABLE 1:** Characteristics of Bordetella pertussis isolates from six school-associated outbreaks in Japan

Outbreak	Location (prefecture)	No. of isolates	Sampling date of 1^st^ isolate^*a*^	Sampling date of last isolate^*a*^	Time range (days)	MLVA^*b*^ type	Virulence-associated allelic genes	SNP^*c*^ genotype^*d*^	Reference
1	Miyazaki	15	11/24/2010	3/14/2011	110	MT27	*ptxP3*/*ptxA1*/*prn2*/*fim3A*	SG2	11, 16
2	Chiba	10	6/24/2012	7/24/2012	30	MT27	*ptxP3*/*ptxA1*/*prn2*/*fim3A*	SG7	This study
3	Toyama	4	11/25/2015	2/22/2016	89	MT27	*ptxP3*/*ptxA1*/*prn2*/*fim3A*	SG10	16
4	Nagano	11	5/23/2016	8/30/2016	99	MT27	*ptxP3*/*ptxA1*/*prn2*/*fim3A*	SG7	16
5	Mie	7	7/22/2016	8/27/2016	36	MT27	*ptxP3*/*ptxA1*/*prn2*/*fim3A*	SG5	This study
6	Niigata	4	5/24/2018	6/6/2018	13	MT27	*ptxP3*/*ptxA1*/*prn9*/*fim3A*	SG10	16

a mm/dd/yyyy.

b MLVA: multilocus variable-number tandem repeat analysis.

c SNP: single-nucleotide polymorphism.

d Simple SNP genotyping with 20 SNP targets.

The 28 sporadic isolates previously genotyped as MT27 carried the virulence-associated genes *ptxP3*, *ptxA1*, *prn2* or *prn9*, and *fim3A* or *fim3B* alleles (Table S1). These isolates belonged to the SNP genotypes SG1, SG2, SG5, SG6, SG7, SG8, and SG10.

### Whole-genome phylogenetic analysis of outbreak and sporadic isolates.

**FIG 1:**
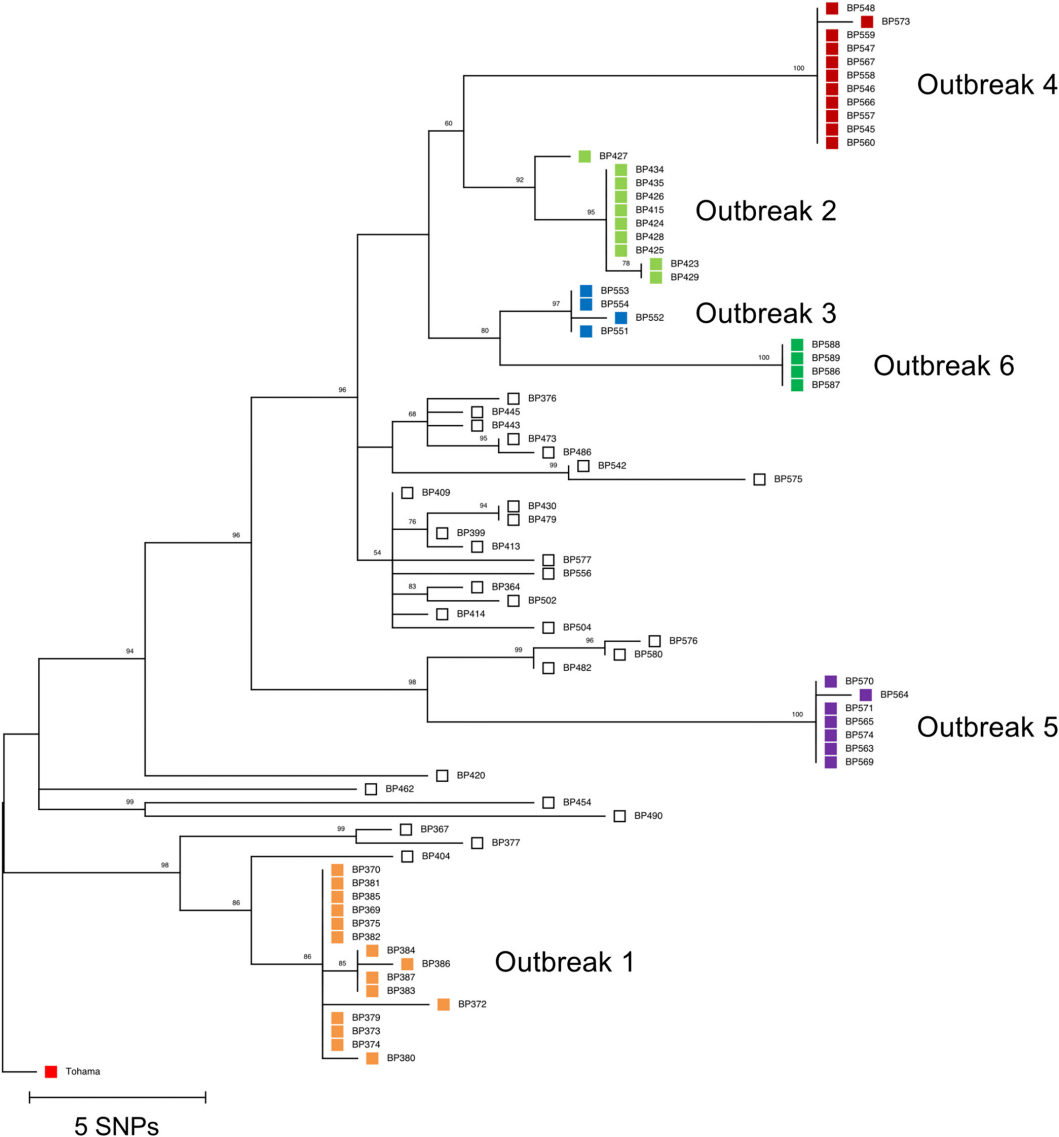
Phylogenetic relationships of Bordetella pertussis isolates from 6 school-associated outbreaks in Japan. The maximum parsimony tree, including 51 outbreak isolates (colored squares) and 28 sporadic isolates (open squares), was constructed based on 169 SNPs with 1,000 bootstrap replicates. B. pertussis Tohama I (red square) served as the reference genome (accession no. NC_002929.2).

Fig. 1 shows the phylogenetic tree of 51 outbreak isolates compared with 28 sporadic isolates based on 169 SNPs. The outbreak isolates were grouped into 6 phylogenetic clusters; the isolates from each outbreak clustered together. No outbreak isolates were phylogenetically unrelated to the outbreaks. The phylogenetic analysis illustrated that outbreak isolates had low SNP diversity (up to 5 SNPs) in each outbreak. In contrast, almost all sporadic isolates (26/28) differed in the phylogenetic tree, but 2 isolates were identical to each other (see Table S4).

The sporadic isolates were grouped into 3 lineages, namely, A, B, and C (Fig. S1). The isolates in lineage A had a higher number of SNP differences than those in lineages B and C; the mean of intra-clade SNP diversity was 23.2, 6.7, and 4.7 in lineages A, B, and C, respectively.

### SNP accumulation rate during outbreaks.

**FIG 2:**
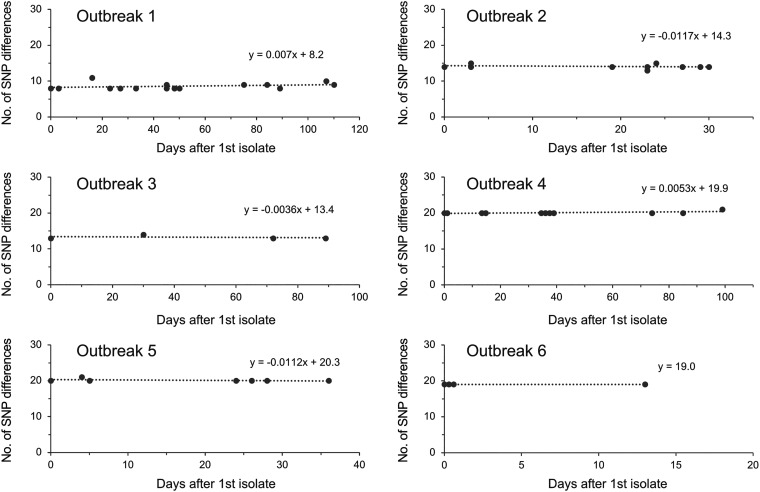
Single-nucleotide polymorphism (SNP) accumulation in Bordetella pertussis during 6 school-associated outbreaks. The number of SNP differences between a particular isolate and the reference strain Tohama I was plotted against the date of specimen collection for each outbreak. The sampling date of the first isolate was counted as day 0 for each outbreak. The dotted lines represent linear regressions.

SNP accumulation rates in the genome of B. pertussis during each outbreak were analyzed (Fig. 2). Among the 15 isolates from outbreak 1, the number of SNP differences (root-to-tip distance) was 8 to 11 during 110 days (Table S2). The linear regression slope was 0.007 SNPs/genome/day, corresponding to 2.56 SNPs/genome/year. Likewise, the SNP accumulation rate was -4.27 SNPs/genome/year for outbreak 2 (30 days), -1.31 for outbreak 3 (89 days), 1.94 for outbreak 4 (99 days), -4.09 for outbreak 5 (36 days), and 0 for outbreak 6 (13 days). The SNP accumulation rates were -4.27 to 2.56 SNPs/genome/year, and the time-weighted average rate was determined to be 0.21 SNPs/genome/year.

### Pairwise SNP differences in the outbreak and sporadic isolates.

Table 2 summarizes the pairwise SNP differences in isolates from each outbreak compared to those in sporadic isolates. Isolate pairs with ≤1 SNP differences were predominant in all outbreaks, and pairs with ≥2 SNP differences were detected only in outbreaks 1 and 2. Overall, the number of SNP differences ranged from 0 to 5 among 238 isolate pairs from 6 outbreaks, and the mean and median values of the number of SNP differences were 0.74 and 0, respectively. On the other hand, among the 378 isolate pairs from the sporadic isolates, the number of SNP differences ranged from 0 to 36, and the isolate pairs with ≥6 SNP differences were predominant (85%). The mean and median values of the number of SNP differences were 16.12 and 17, respectively.

**TABLE 2:** Pairwise single-nucleotide polymorphism (SNP) differences in outbreak isolates and sporadic isolates of Bordetella pertussis

			No. of isolate pairs with									No. of pairwise SNP^*a*^ differences	
Case	No. of isolates	No. of isolate pairs	0 SNP	1 SNP	2 SNPs	3 SNPs	4 SNPs	5 SNPs	6−10 SNPs	11−20 SNPs	21−36 SNPs	Mean/Median	Range
Outbreak 1	15	105	39	39	12	10	4	1				1.09/1	0−5
Outbreak 2	10	45	22	14		7	2					0.96/1	0−4
Outbreak 3	4	6	3	3								0.50/0.5	0−1
Outbreak 4	11	55	45	10								0.18/0	0−1
Outbreak 5	7	21	15	6								0.29/0	0−1
Outbreak 6	4	6	6									0/0	0
Total	51	238	130	72	12	17	6	1				0.74/0	0−5
Sporadic	28	378	1	5	7	14	14	17	80	96	144	16.12/17	0−36

a SNP: single-nucleotide polymorphism.

### Determination of an optimal SNP threshold.

**FIG 3:**
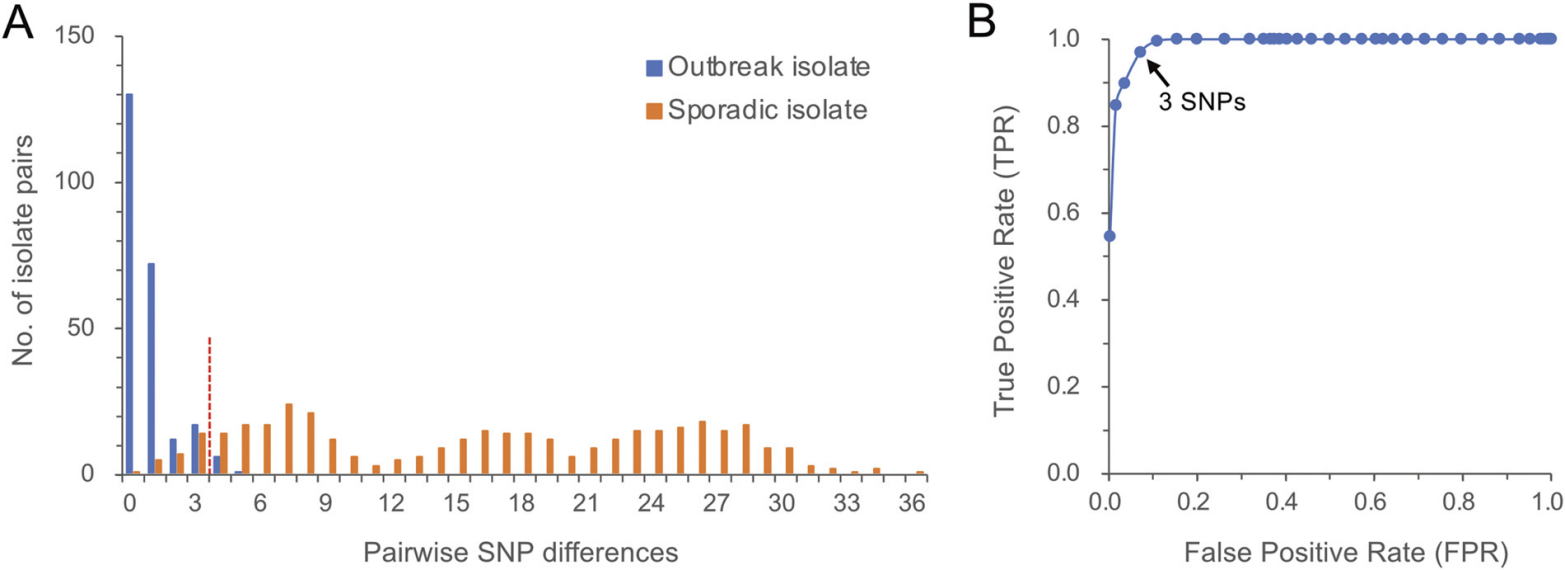
Determination of an optimal single-nucleotide polymorphism (SNP) threshold for distinguishing between Bordetella pertussis outbreak and sporadic isolates. (A) Distribution of the number of pairwise SNP differences in B. pertussis outbreak and sporadic isolates. A total of 238 pairs of outbreak isolates were compared with 378 pairs of sporadic isolates. (B) Receiver operating characteristic (ROC) curve for determining an optimal SNP threshold. The arrow points to the highest Youden’s index of 0.90, corresponding to 3 SNP differences between isolate pairs.

Figure 3A shows the distribution of pairwise SNP differences in the outbreak and sporadic isolates. The distribution of sporadic isolates partially overlapped with the distribution of outbreak isolates: 58 (15%) of the 378 pairs of sporadic isolates overlapped in 0 to 5 SNP differences. At the cutoff value of 3 SNPs, the TPR was 0.97 (correctly identified outbreak isolate as an outbreak isolate), and FPR was 0.07 (incorrectly identified sporadic isolate as an outbreak isolate) (Fig. 3B). The cutoff value yielded the highest Youden’s index (*J*) of 0.90 (Table S5). The receiver operating characteristic (ROC) curve and Youden’s index identified 3 SNPs as an optimal threshold for distinguishing between outbreak and sporadic isolates.

## DISCUSSION

In the present study, B. pertussis isolates from 6 school-associated outbreaks exhibited low SNP diversity during the outbreaks. The mean SNP accumulation rate was estimated to be 0.21 SNPs/genome/year (time-weighted average), and the optimal SNP cutoff value for distinguishing between outbreak and sporadic isolates was determined as 3 SNPs. Here, we propose a genetic threshold of ≤3 SNPs per genome as a marker of strain identity for detecting and investigating pertussis outbreaks. To the best of our knowledge, this study is the first to establish a genome-based SNP threshold for B. pertussis epidemic strain MT27.

Based on the distribution of the number of pairwise SNP differences between the outbreak and sporadic isolates, we defined ≤3 SNPs as a threshold for the same outbreak strain: the threshold exhibited the highest Youden’s index of 0.90 with a TPR of 0.97 and an FPR of 0.07. However, a change in SNP diversity among sporadic isolates may affect the SNP threshold. Here, our sporadic isolates exhibited a trimodal distribution for the number of pairwise SNP differences instead of a normal distribution (Fig. 3A). The sporadic MT27 isolates were grouped into 3 lineages (A, B, and C), and the isolates in lineage A had a higher number of SNP differences than those in other lineages, likely causing the trimodal distribution of the number of SNP differences (Fig. S1). Our previous study demonstrated that the frequency of strain SG1 in lineage A decreased in 2010 in Japan and the US, whereas that of strain SG7 in lineage C markedly increased (16). This observation implies that SNP diversity in B. pertussis MT27 strains may be lowered by a large increase in strain(s), such as SG7. The decrease in SNP diversity might affect the SNP threshold defining strain identity, and thus continuous genome surveillance on the circulating B. pertussis strains is required for accurate detection and investigation of outbreaks.

In our school-associated outbreaks 1 and 2, two isolates exhibited a higher number of SNP differences than the other isolates: the isolate BP372 from outbreak 1 had 3 to 5 SNP differences, and the isolate BP427 from outbreak 2 had 3 to 4 SNPs (Fig. 1 and Table S3). The BP372 was isolated from a 14-year-old girl at a middle school where the outbreak occurred, and the BP427 was isolated from a 4-year-old girl at the same clinic as the other patients. One possible explanation for the higher number of SNP differences is the intra-clonal SNP diversity of B. pertussis in a single patient. A previous study demonstrated that multiple isolates collected from individual patients had up to 5 SNP differences in a single patient (15). Another possible explanation is the time-dependent accumulation of SNPs in B. pertussis. Previous school outbreak investigations showed the presence of asymptomatic carriers for several months before the outbreak was detected (9, 10). School-associated outbreaks occur over a longer period, potentially leading to a higher number of SNP differences in individual isolates from a single outbreak.

In previous studies, the mutation rate of B. pertussis was estimated to be 3.06 × 10^−6^–3.81 × 10^−7^ substitutions per site per year, which was determined using isolates collected from various locations and years (13, 18, 31, 32). This mutation rate was calculated as 0.9 to 12.6 SNPs/genome/year using the average genome size of B. pertussis, i.e., 4.1043 Mb (NCBI genome database). In this study, the SNP accumulation rate of the outbreak strain (epidemic MT27 strain) was 0.21 SNPs/genome/year (95% confidence interval [CI], -1.80 to 2.22), which is lower than those reported in the above-mentioned studies. Excluding the short-term outbreaks 2, 5, and 6 (time ≤ 36 days) from the calculation, the SNP accumulation rate was estimated to be 1.19 SNPs/genome/year (95% CI, -0.69 to 3.07), indicating that our SNP accumulation rate was lower due to a bias created by the short-term outbreaks. Therefore, we could not verify the SNP threshold based on the SNP accumulation rate. Further studies using isolates from long-term outbreaks are needed to assess the SNP accumulation rate in B. pertussis epidemic strain MT27.

Here, we found that 2 SNPs (G667028A and C4068650T, reference positions in the Tohama genome) were the primary characteristic of sporadic isolates (Table S2). The G667028A, which results in a nonsense mutation (Gln30Stop) in NAD(P)/FAD-dependent oxidoreductase, is present in nearly all sporadic isolates (93%), but not in all outbreak isolates. Similarly, the majority of sporadic isolates (93%) carry the C4068650T mutation, which results in a non-synonymous substitution (Gly295Ser) in ATP-binding cassette domain-containing protein. The SNPs were identified in previous studies; however, their specificity toward the isolated sporadic was unknown (16, 33). Additionally, to assess the impact of the SNPs on the virulence of outbreak and sporadic strains, further studies are needed.

Further, to validate our proposed SNP threshold, we analyzed SNP differences in 12 outbreak isolates collected from a high school in the US using publicly available genome sequences; the outbreak spanned <2 months (15). The outbreak isolates exhibited 0 to 2 SNP differences (mean, 0.61; median, 1), thereby confirming the validity of our threshold of ≤3 SNPs. However, here we analyze only B. pertussis isolates from school-associated outbreaks that less than 4 months. A limitation of this study is that it is uncertain whether our SNP threshold can be applied to isolate(s) from long-term outbreaks (4 months or more).

In conclusion, the present study describes the optimal SNP threshold defining strain identity for pertussis outbreak investigations. Recently, WGS has emerged as an important tool for genotyping B. pertussis because of its higher discriminatory power compared to that of classical PCR-based genotyping methods, such as MLVA and MLST. To the best of our knowledge, this study provides the first reliable threshold for genome-wide SNP analysis to detect and investigate pertussis outbreaks.

## MATERIALS AND METHODS

### Outbreak and sporadic isolates.

In this study, 51 outbreak isolates of B. pertussis were investigated. They were isolated from 51 patients infected during 6 school-associated outbreaks that occurred in the 2010s in rural areas of Japan: 15 isolates from Miyazaki prefecture, 10 from Chiba, 4 from Toyama, 11 from Nagano, 7 from Mie, and 4 from Niigata (Table 1) (11, 16). Most isolates (47/51) were collected from children studying in elementary and middle schools, and the remaining were from their siblings and school officials. Isolation of B. pertussis was performed on Bordetella CFDN agar plates (Nikken Bio Medical Laboratory) using nasopharyngeal swab specimens. Thereafter, the isolates were cultured on Bordet-Gengou agar or Bordetella CFDN agar plate at 36°C for 2 to 3 days and bacterial DNA was extracted. Further, 28 sporadic isolates collected in Japan in the 2010s were compared with the outbreak isolates. The sporadic isolates were selected from the National Institute of Infectious Diseases (NIID) strain collection to reflect the same MLVA genotype as the outbreak isolates (genotype MT27). Detailed information on the outbreak and sporadic isolates is provided in Table S1.

### Genotyping.

PCR-based genotyping was performed using multilocus antigen sequence typing (MAST) targeting *ptxP*, *ptxA*, *prn*, and *fim3* alleles, multilocus variable-number tandem repeat analysis (MLVA) targeting 6 VNTR loci, and simple SNP genotyping with 20 SNP targets (4, 16, 34, 35).

### WGS.

Genomic DNA was purified from outbreak isolates by the NucleoSpin Tissue kit (Macherey-Nagel), and samples were sequenced on the Illumina NovaSeq 6000, or HiSeq X 10 platform with 150-bp paired-end reads. Library preparation and sequencing on the platforms were performed by Novogene. The average coverage depth of the sequencing exceeded 290× for each isolate. Sporadic isolates were sequenced in previous studies (16, 17). Sequencing information in this regard is summarized in Table S1.

### SNP calling, phylogenetic analysis, and pairwise SNP difference.

Illumina short-read data were assembled *de novo* using CLC Genomic Workbench version 20.0.4 (CLC Bio) set to default. For SNP calling, contigs were mapped to the reference genome sequence of B. pertussis Tohama I (accession no. NC_002929.2) using CSI Phylogeny 1.4 (36) set to default. The accuracy of SNP calls was manually inspected for mapped reads to the reference genome using the CLC Genomic Workbench, and a total of 169 high-quality SNPs were identified among the outbreak and sporadic isolates (Table S2). A maximum parsimony tree with 1,000 bootstrap replicates was constructed based on the 169 SNPs using MEGA X version 10.1.8 with default settings (37). Pairwise SNP differences were calculated for each outbreak using the MEGA X (Table S3).

### ROC analysis.

ROC analysis was performed to determine an optimal SNP cutoff value for distinguishing between outbreak and sporadic isolates. The true-positive rate (TPR, sensitivity) and false-positive rate (FPR, 1 - specificity) were calculated by pairwise SNP differences, and Youden’s index (*J*) was calculated using the formula *J* = TPR − FPR. This index was used to determine an optimal SNP threshold.

### Data availability.

All genome sequence data generated as part of this study were submitted to the DDBJ Sequence Read Archive (SRA) under the BioProject number PRJDB7578. The SRA accession numbers are listed in Table S1.
