# Supplementary material for: Whole-Genome Analysis of Bordetella pertussis MT27 Isolates from School-Associated Outbreaks: Single-Nucleotide Polymorphism Diversity and Threshold of the Outbreak Strains
Source: Microbiol Spectr. 2023 May 16;11(3):e04065-22. doi: 10.1128/spectrum.04065-22 (PMC10269452; doi:10.1128/spectrum.04065-22)
Supplement: Supplemental file 1 — Fig. S1. Download spectrum.04065-22-s0001.pdf, PDF file, 0.3 MB [file spectrum.04065-22-s0001.pdf]

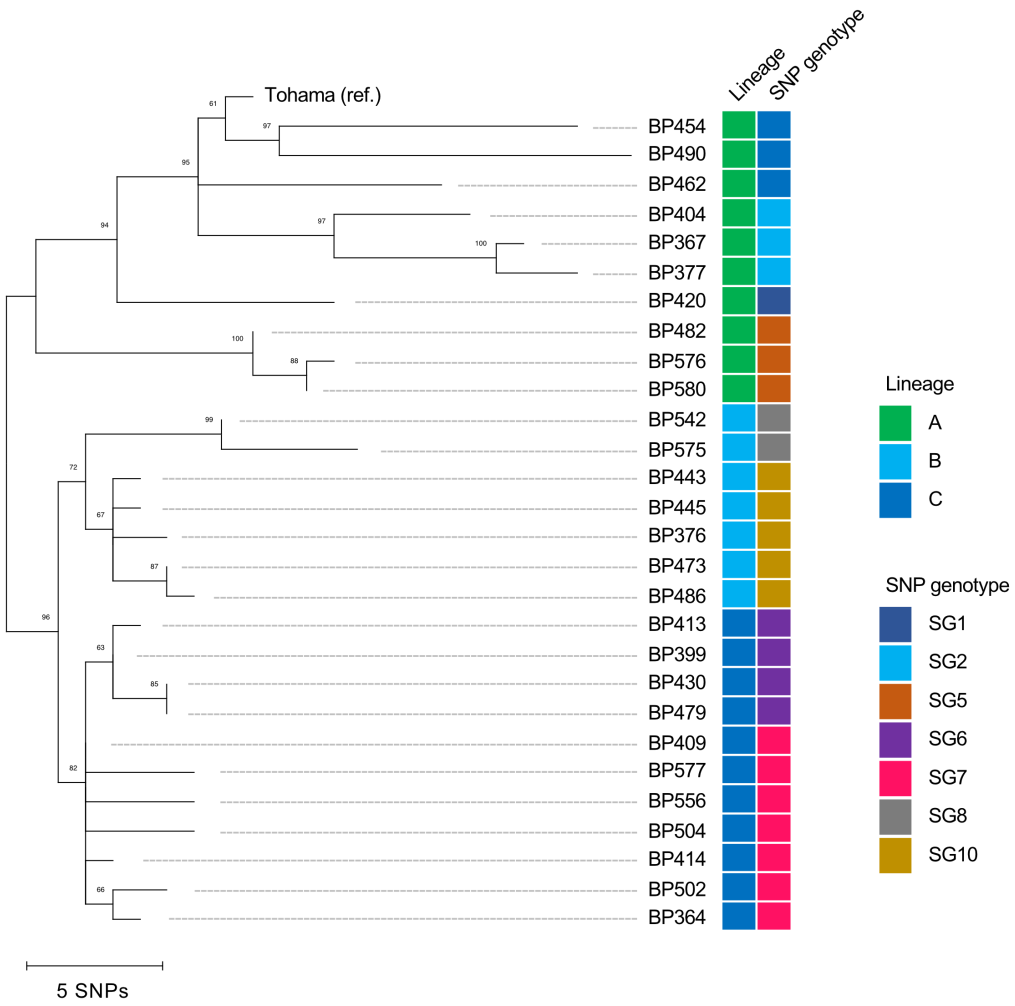

FIG S1 Phylogenetic relationship of 28 sporadic isolates of *Bordetella pertussis*. The maximum parsimony tree was constructed based on 125 SNPs with 1,000 bootstrap replicates. *B. pertussis* Tohama I served as the reference genome (accession no. NC_002929.2). The SNP genotypes were determined by the simple SNP genotyping with 20 SNP targets.
